# Supplementary material for: A Deep Learning-Aided Automated Method for Calculating Metabolic Tumor Volume in Diffuse Large B-Cell Lymphoma
Source: Cancers (Basel). 2022 Oct 25;14(21):5221. doi: 10.3390/cancers14215221 (PMC9653575; doi:10.3390/cancers14215221)
Supplement: Supplementary file 1 [file cancers-14-05221-s001.zip › cancers-1904973-supplementary.pdf]

Supplemental Data

**Supplemental Table S1**. Concordance between readers in SUVmax values by scanner type				
**Sample**	**N**	**Reader 1 vs Reader 2**	**Reader 1 vs AM**	**Reader 2 vs AM**
**All**	100	ICC=1 p=<.0001 (95%CI= 1 to 1)	ICC=1 p=<.0001 (95%CI=0.99 to 1)	ICC=1 p=<.0001 (95%CI=0.99 to 1)
**GE**	30	ICC=1 p=<.0001 (95%CI= 1 to 1)	ICC=1 p=<.0001 (95%CI=0.99 to 1)	ICC=1 p=<.0001 (95%CI=0.99 to 1)
**Philips**	17	ICC=1 p=<.0001 (95%CI= 1 to 1)	ICC=0.81 p=<.0001 (95%CI=0.57 to 0.93)	ICC=0.81 p=<.0001 (95%CI=0.57 to 0.93)
**Siemens**	53	ICC=1 p=<.0001 (95%CI= 1 to 1)	ICC=1 p=<.0001 (95%CI= 1 to 1)	ICC=1 p=<.0001 (95%CI= 1 to 1)
**Abbreviations**; SUVmax, maximum standardized uptake value; AM, automated method; ICC, inter-class correlation coefficients; 95%CI, 95% confidence intervals.				

**Supplemental Table S2**. Concordance between readers in MTV values by scanner type				
**Sample**	**N**	**Reader 1 vs Reader 2**	**Reader 1 vs AM**	**Reader 2 vs AM**
**All**	100	ICC=1 p=<.0001 (95%CI= 1 to 1)	ICC=0.98 p=<.0001 (95%CI=0.96 to 0.99)	ICC=0.98 p=<.0001 (95%CI=0.96 to 0.99)
**GE**	30	ICC=1 p=<.0001 (95%CI= 1 to 1)	ICC=0.96 p=<.0001 (95%CI=0.90 to 0.98)	ICC=0.96 p=<.0001 (95%CI=0.90 to 0.98)
**Philips**	17	ICC=1 p=<.0001 (95%CI= 1 to 1)	ICC=1 p=<.0001 (95%CI=0.96 to 1)	ICC=1 p=<.0001 (95%CI=0.96 to 1)
**Siemens**	53	ICC=1 p=<.0001 (95%CI= 1 to 1)	ICC=0.98 p=<.0001 (95%CI=0.96 to 0.99)	ICC=0.98 p=<.0001 (95%CI=0.96 to 0.99)
**Abbreviations**; MTV, metabolic tumor volume; AM, automated method; ICC, inter-class correlation coefficients; 95%CI, 95% confidence intervals.				

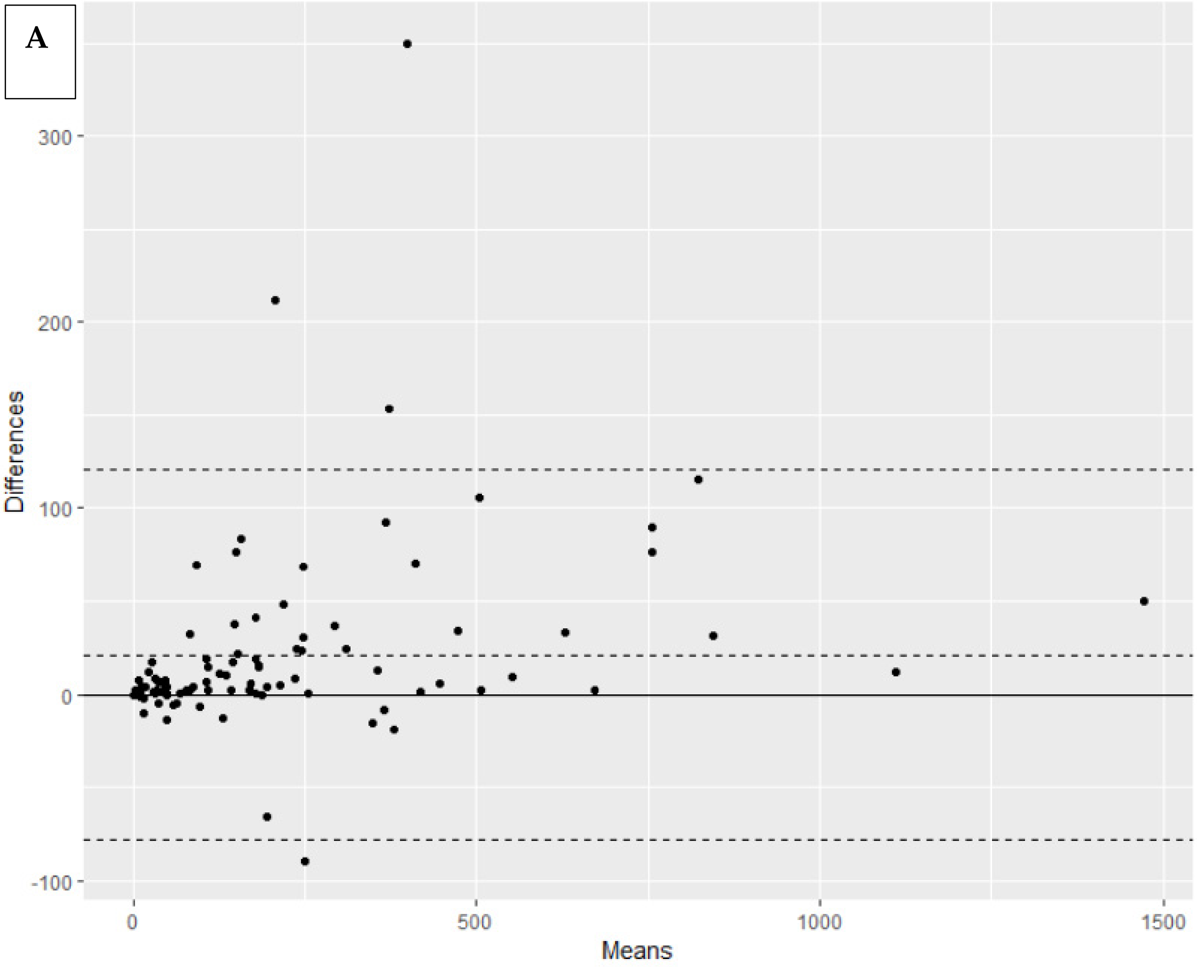
A
Differences
Means
300
200
100
0
-100
0
500
1000
1500

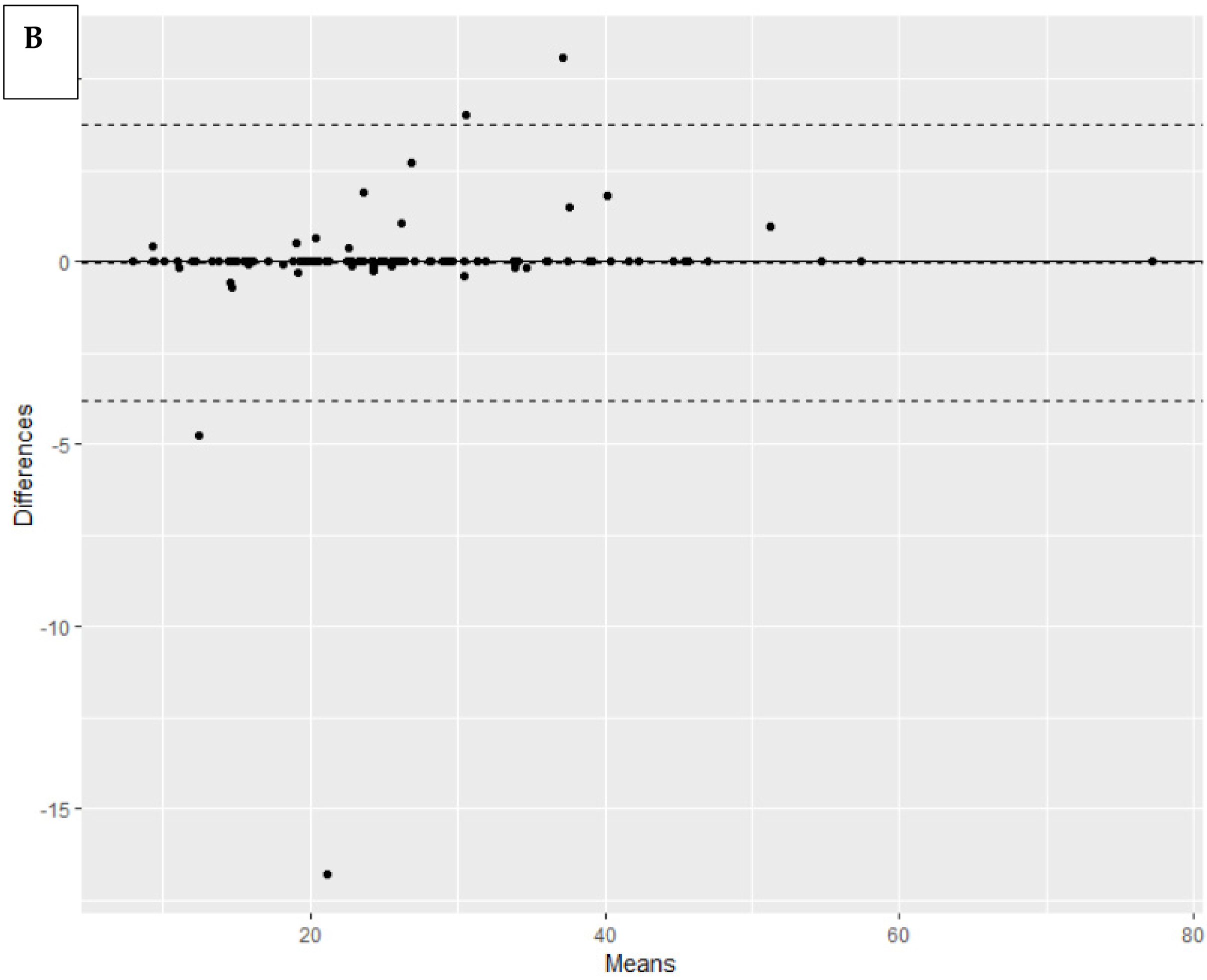

Supplemental Figure S1. (A). Bland-Altman plot. Graphical display for bias and Root-Mean-Squared Error (RMSE) between average of reader 1 and reader 2 versus automated method in metabolic tumor volume calculations. (B) Bland-Altman plot. Graphical display for bias and Root-Mean-Squared Error (RMSE) between average of reader 1 and reader 2 versus automated method in SUVmax calculations.
